# Microbial Food Safety Risk to Humans Associated with Poultry Feed: The Role of Irradiation

**DOI:** 10.1155/2019/6915736

**Published:** 2019-01-22

**Authors:** Tahiru Mahami, Wellington Togby-Tetteh, Delali Isaac Kottoh, Leticia Amoakoah-Twum, Emmanuel Gasu, Sylvester Nana Yao Annan, Daniel Larbi, Isaac Adjei, Abraham Adu-Gyamfi

**Affiliations:** Biotechnology and Nuclear Agriculture Research Institute (BNARI), Ghana Atomic Energy Commission, P. O. Box LG 80, Legon, Accra, Ghana

## Abstract

Animal feed has been linked to human illness through the food chain as a result of food borne bacteria and more recently the risk of foodborne antibiotic resistance. This study investigated the extent to which radiation can be used as an intervention to improve the safety and quality of poultry feed in terms of food borne pathogens and antibiotic resistant microbes. Mean counts of control feed samples were Log_10_ 5.98 for total viable count (TVC), Log_10_ 4.76 for coliform count (CC), Log_10_ 2.89 for* Staphylococcus aureus* count (STC), and Log_10_ 4.57 for yeast and mold count (YMC) and* Salmonella *spp. (SC) was not detected (ND). All counts were within permissible levels except for CC (Log_10_ 4.76) which was above the permissible limit of ≤ log_10_ 4.0. Identified bacteria isolates were* Enterobacter cloacae* (54.5%),* Bacillus cereus* (27.3%), and* Klebsiella pneumoniae* (18.2%). All (100%) isolates exhibited multidrug Resistance (MDR) with* Bacillus cereus* being the most resistant (to 9 out of 11 antibiotics) followed by* Enterobacter cloacae/Klebsiella pneumoniae *(4 out of 11 antibiotics). Several resistance patterns were observed with PEN/AMP/FLX being the commonest (100%), followed by ERY (90.9%), TET (72.7%), CRX (66.6%), CTX (45.4%), CHL/CTR (36.4%), GEN (27.3%), and COT (18.2%).* Klebsiella pneumoniae* showed zero resistance to GEN/CHL/CTR/CTX/CRX while* Enterobacter cloacae *and* Bacillus cereus *exhibited zero resistance to GEN and COT, respectively. The most effective antibiotic against Gram negative bacteria (*Enterobacter cloacae *and* Klebsiella pneumoniae) *was gentamicin while cotrimoxazole was the most effective against* Bacillus cereus* (Gram positive). Radiation processing of 5kGy totally eliminated all microbes including MDR food borne pathogens. In view of this, we recommend low dose radiation decontamination as a measure to mitigate against the possible food safety and public health risks to humans associated with poultry feed.

## 1. Background

Animal feed has been known to contribute to the disease burden of man via the food chain [1]. Concern about the food safety risk to humans associated with animal feed however gained prominence only when Creutzfeldt-Jakob (‘mad cow') disease was first described in humans [1, 2]. Contaminated poultry products contribute significantly to food-borne bacterial diseases (FBD) [3, 4], the impact of which is significantly linked to high morbidity and mortality globally [5]. Economic impact of food safety outbreaks on food businesses and the effect of food borne diseases on a nation's economy has been documented (Hussain et al., 2018). Evidence specifically establishes that when animals reared for their meat are colonized by Salmonella and other bacteria pathogens, these pathogens can be spread to humans via the food chain [1, 6]. Unfortunately, the food-borne bacterial pathogen risk to humans associated with poultry feed has not been given the desired attention. Current drivers of FBDs like emergence of new pathogens [7], emergence of antibiotic resistant bacteria, a growing size of immunocompromised population [5] and a change in cooking habits call for more attention to this FBD risk associated with poultry feed.

Seventy percent of animal feed produced in Ghana is poultry feed which is either manufactured by commercial feed millers or on-farm self-millers [8]. Even though different sources of feed present different levels of risk of microbial contamination, generally contamination occurs along the feed production value chain and it is almost impossible to produce sterile feed. Good manufacturing practices, postproduction decontamination, and suitable facilities for storage are therefore required to improve the safety and shelf-life of feed.

Conventional methods applied by feedmills in eliminating bacteria contaminants in feed are limited particularly in the control of spore formers [9]; hence antimicrobial drugs are used to improve feed safety and shelf-life but this selects for antimicrobial resistant bacteria which then serve an additional risk to humans through the food chain [10]. An alternative to conventional methods is irradiation of feed using gamma rays from a Cobalt-60 source. Predetermined doses of radiation can be used as an alternative for food and food products decontamination that will improve safety, quality, and shelf-life [11]. Irradiation totally eliminates salmonellae, enterobacteria, molds, and insects form feed [11] and improves the utilization and digestibility of proteins and carbohydrates by breaking them down into easily digestible forms [12, 13].

Unfortunately, there is no comprehensive program in most developing countries in general and Ghana in particular that addresses animal feed contamination in food safety programs and radiation processing of poultry feed has not been explored in Ghana to avail to the poultry industry and the public health sector the immense benefits thereof. The objective of the current study was mainly to investigate the effect of gamma radiation on the microbial quality of compounded poultry feed.

## 2. Materials and Methods

### 2.1. Sampling and Sample Preparation

Antibiotic-free compounded feed (made of corn, wheat bran and soy bean) was collected from the Biotechnology and Nuclear Agriculture Research Institute (BNARI) poultry unit aseptically into sterile zip polyethylene bags and sent to the laboratory for analysis. In the laboratory, subsamples were aseptically weighed from the main sample into smaller polyethylene bags (100g in each bag) in duplicate, labeled according to the radiation doses (0, 5, 10, 15, 20, and 25 kGy), and sealed for irradiation.

### 2.2. Irradiation

The radiation doses 5, 10, 15, 20, and 25 kGy from a Cobalt-60 source (SLL-02, Hungary) were applied to samples at the Radiation Technology Centre of Ghana Atomic Energy Commission. The absorbed dose was confirmed by dosimetry.

### 2.3. Microbial Analysis

#### 2.3.1. Determination of Microbial Load and Identification of Isolates

International Organization for Standardization (ISO) methods were modified and adopted for the enumeration of total viable count (TVC), coliform count (CC), and yeast and molds count (YMC). For (TVC) the temperature for incubation of ISO 4833:2003 was modified from 30°C to 35°C and plate count agar (PCA) was used. ISO 21528-2:2004 was modified and applied for the enumeration of general coliforms. The incubation temperature was also modified to 35°C and Eosin Methylene Blue (EMB) agar was used. Oxytetracycline Glucose Yeast Extract (OGYE) agar was used for the enumeration of yeast and molds at an incubation temperature of 28°C (a modification of ISO 21527-1). Baird Parker agar (Oxoid, UK) was used for the* Staphylococcus aureus *count at 35°C incubation and representative colonies were confirmed after 24hrs by the coagulase test using the staphylase kit (Oxoid, UK). 

Media were all prepared and sterilized according to manufacturer's instructions, kept molten at 45°C in a water bath and poured aseptically over serially diluted dispensed samples in sterile petri dishes (20ml per dish). Each dilution was plated in duplicate. Petri dishes were gently swirled to mix and media were allowed to set after which they were incubated with lid of petri dish downwards to prevent backflow of moisture into the culture. Colonies were counted after 48 hours with a colony counter and the CFU/g calculated. Specific pathogens were also identified following laboratory methods of plating and isolation. API 20E kits (bioMérieux, France) was used to identify Gram negative bacteria while conventional biochemical methods described by [14] were used to identify Gram positive bacteria.

#### 2.3.2. Antibiotic Susceptibility Testing

Procedures were first described by [15] and adopted by CLSI [16] (2 were followed in determining antibiotic sensitivity). Twenty-four-hour pure cultures of identified isolates were made on nutrient agar to produce enough growth. Inoculum of isolated strains was then prepared by inoculating aseptically a universal bottle containing 9ml of 0.1% sterile peptone water using a wire loop. The concentration of inoculum was standardized by adjusting its turbidity to 0.5 McFarland standards. Inoculum was applied onto the surface of prepared and dispensed (into petri dishes). Mueller-Hinton sensitivity agar used a calibrated wire loop. A sterile cotton swab was then used to spread the culture on the surface of the media. The inoculated plate was allowed to dry for some few minutes after which commercially procured sensitivity disks (Oxoid, UK) were applied to it using a sterile forceps. Zones of inhibition around sensitivity disks were measured to the nearest millimeter using a pair of calipers after 18-24hr of incubation at 37°C and results recorded. The interpretive criteria (zone diameter values) of CLSI [16] were used to indicate susceptible, intermediate, and resistant breakpoints.

The following antibiotics were used: Beta lactams-Penicillin's (penicillin 10*μ*g, Ampicillin 10*μ*g, and Flucloxacillin 5*μ*g) and Cephalosporins (Cefuroxime 30*μ*g, Cefotaxime 30*μ*g, and Ceftriaxone 30*μ*g), Tetracyclines (Tetracycline 10*μ*g), Macrolides (Erythromycin 15*μ*g), Aminoglycosides (Gentamicin 10 *μ*g), Sulfonamides (Cotrimoxazole 25 *μ*g/disk), and Chloramphenicol 30 *μ*g/disk.

### 2.4. Data Analysis

Microbial counts in colony forming units (cfu/g) were determined following standard formulae and converted to logarithms (log10).

## 3. Results

The mean counts of total viable cells, coliforms, Staphylococcus, and yeast and molds of compound feed were, respectively, 5.98, 4.76, 2.89, and 4.57 log_10_cfu/g.* Salmonella *spp. were not detected. Details of the effect of radiation doses applied on these microbial populations are summarized in Table 1.

The results (Table 1) show that all viable cells counted were all within permissible levels of contamination except for coliform count (log_10_4.76) which was higher than the permissible level of ≤ log_10_ 4.0. 5KGy of gamma radiation totally eliminated all viable cells of all kinds and making contamination levels acceptable (Table 1). 

Results of bacterial identification yielded 11 bacterial isolates which were identified into 3 genera: Bacillus, Enterobacter, and Klebsiella. Details of bacteria genera occurrence in nonirradiated feed samples are presented in Table 2.

*Enterobacter cloacae* was the most common bacteria species identified (54.5%) followed by* Bacillus cereus* (27. 3%) and then* Klebsiella pneumoniae* (18.2%) (Table 2).

**Figure 1:**
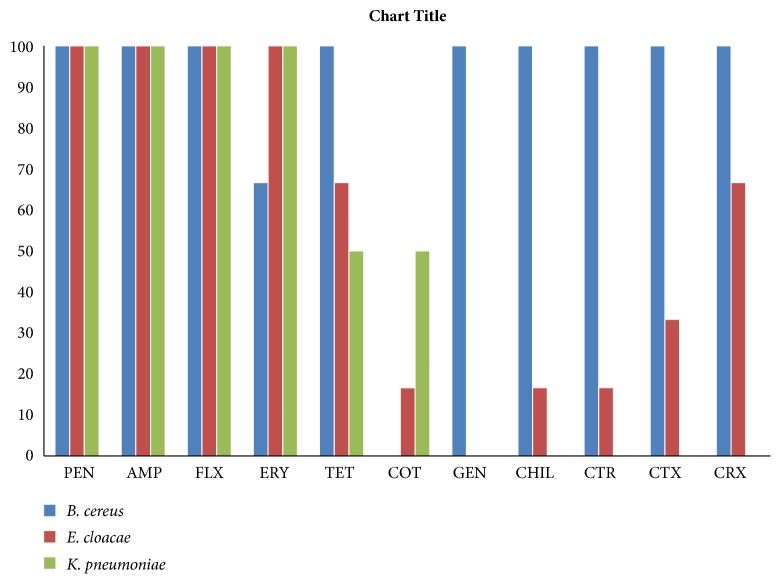
**Resistance of identified isolates to antimicrobial agents.** PEN= Penicillin, AMP= Ampicillin, FLX= Flucloxacillin, ERY= Erythromycin, TET=Tetracycline, COT= Cotrimoxazole, GEN=Gentamicin, CHL=Chloramphenicol,, CTR= Ceftriaxone, CTX= Cefotaxime, and CRX= Cefuroxime.

Results (Figure 1) reveal that all isolates were MDR, with* B. cereus* being the most resistant (resistant to 9 out of 11) followed by* Enterobacter cloacae/Klebsiella pneumoniae *(resistant to 4 out of 11 antibiotics). The most effective antibiotic against Gram negative bacteria (*Enterobacter clocae* and* Klebsiella pneumoniae) *was gentamicin while cotrimoxazole was the most effective against* Bacillus* cereus (Gram positive) (Figure 1).

Antibiotic resistance patterns of identified bacteria isolates from antibiotic-free compounded feed are presented in Table 3

The results (Table 3) show that eight resistance patterns were exhibited with PEN/AMP/FLX being the commonest (100%), followed by ERY (90.9%), TET (72.7%), CRX (66.6%), CTX (45.4%), CHL/CTR (36.4%), GEN (27.3%), and COT (18.2%).* Klebsiella pneumoniae* showed zero resistance to GEN/CHL/CTR/CTX/CRX while* Enterobacter cloacae *and* Bacillus cereus *exhibited zero resistance to GEN and COT, respectively (Figure 1).

## 4. Discussions

The current study found all feed samples to be contaminated with viable microbes but counts were within permissible levels except for coliform count which was above the permissible level of ≤ log_10_ 4.0 (Table 1). No Salmonella was however detected by the methods used (Table 1). This finding agrees with the general view that poultry feed is prone to microbial contamination along the value chain, from the environment, during transport, and storage on the farm and cross contamination from wild birds, insects, rodents, etc. [18, 19].

Both total viable count and yeast and mold counts are indicators of quality of a food product and have more to do with determining the shelf-life of a product [20] A total viable count of >10^7^CFU/g) is said to hasten spoilage or deterioration of the product [21]. Permissible levels of total viable counts and yeast and mold count observed in this study (Table 1) suggest that good manufacturing practices, handling conditions, and storage conditions may have been observed.* Staphylococcus aureus* is also an indicator of the hygiene of a product as this organism is part of the microflora of both chickens and human [22]. Ingestion of the thermostable enterotoxins, rather than the bacterium itself, is responsible for foodborne illness [23]. Acceptable levels of* Staphylococcus aureu*s count observed in this study may have also been due to low rate of cross contamination from human handlers of feed and from the farm environment.

Coliform count (Log_10_ 4.76) was observed to be above the permissible limit of ≤ log_10_ 4.0 (Table 1) and this was confirmed by a high occurrence of identified coliforms {*Enterobacter cloacae* (54.5%) and* Klebsiella pneumoniae* (18.25%)} among isolates (Table 2). This finding suggested a poor hygiene status of the feed as a result of possible fecal contamination. Coliform organisms naturally reside in the gut of animals and man and are found on food substances only as a result of fecal contamination due to poor hygiene. Fecal coliform test is therefore a good indicator for the evaluation of hygiene whiles the general coliform test includes coliforms like* Klebsiella *spp. and* Enterobacter *spp. which are also environmental microbes [24]. Cross contamination from hands of farm workers during mixing of feed, from rodents and reptiles on the farm, from droppings of live birds, or from dust or the environment, may have accounted for the unacceptable coliform count recorded in this study. According to [25] the initial microbial load of feed, the sanitization process applied, climatic conditions during storage, and postsanitization handling play a major role in the safety and quality of the final product 

Identified isolates (Table 2) confirms the foodborne pathogen risk associated with feed as all identified organisms are capable of causing human disease.* Enterobacter cloacae* is hospital-acquired and it contributes to the following ailments: bacteremia, endocarditis, septic arthritis, osteomyelitis, skin/soft tissue infections, and lower respiratory tract infections [26] while* Klebsiella pneumoniae* causes infections such as urinary tract infection, pneumonia, intra-abdominal infection, bloodstream infection, meningitis, and pyogenic liver abscess [27].* Bacillus cereus* is a soil bacterium that easily contaminates several food substances such as eggs, meat, dairy, and plant products and is known for causing 25 % of food-borne intoxications due to its secretion of emetic toxins, enterotoxins, and resistance of its spores to heat treatment [28].

Results of antibiotic sensitivity test (Figure 1) demonstrated that all identified isolates were MDR and* Bacillus cereus* was the most resistant isolate (resistant to 9 out of 11 antibiotics) followed by* Enterobacter cloacae/Klebsiella pneumoniae *which were resistant to 4 out of 11 antibiotics. In a similar study, Donkor et al. [29] found 97.7% MDR in* E. coli* isolates from animals in the Accra metropolis. Multidrug resistance occurs in bacteria due to the aggregation of resistance genes from other bacteria with each gene coding for a different antibiotic [5, 30]. Evidence suggests that, among “wild type” bacterial communities, resistance to any type of antibiotic is lower than in clinical isolates or isolates from a source that has been exposed to the use of antibiotics. misuse and overuse of antibiotics due to lack of regulation and proper policies in most developing countries including Ghana especially in agriculture sector could have accounted for this high level of MDR observed in this study (Figure 1).

Beta lactamase producers such as* B. cereus* are intrinsically resistant to *β*-lactam antibiotics [31]; this may have accounted for the high rate of resistance observed to wide-ranging cephalosporins among* B. cereus* isolates in this study (Figure 1). Additionally, the secretion of *β*-lactamase by individual bacteria in a polymicrobial environment also provides passive resistance for all residents within the environment [32] and this may have accounted for high level of MDR among isolates.

The overuse or unregulated use of antibiotics in agriculture is a problem of global concern which is liked to food-borne antibiotics resistance in humans. For example, of all antibiotics sold in the United States, about 80% are applied in animal agriculture out of which, unfortunately, approximately 70% of these are important to human medicine [33]. Antibiotics are used specifically in animal farming to treat diseases and also as growth promoters to enhance growth. Options for treatment of patients with MDR infections are often extremely limited leading to possible treatment failure with enhanced morbidity and mortality and high medical costs [34]. High level of MDR observed in this study is therefore worrying.

Antibiotic susceptibility patterns (Table 3) revealed that common antibiotics used in the treatment of human diseases are generally losing their efficacy on bacteria isolates which is very worrying. For example, Penicillin/Ampicillin/Flucloxacillin (all beta-lactams) which is the most widely used class of antibiotics in humans [31] was not effective against any of the isolates (Table 3). Even though* Enterobacter cloacae* and* Klebsiella pneumoniae* are intrinsically resistant to some beta lactams [16], the level of MDR observed in this study is worrisome.

WHO [35] recommends that antibiotics critically important for humans (Aminoglycosides, 3^rd^ and 4^th^ generation Cephalosporins, and Macrolides) and those highly important for humans (Cephalosporins, Penicillins, and sulfanomides) should not be used for prevention of animal diseases or as growth promoters so that these banned drugs will remain effective in treating human bacterial diseases. Only Tetracyclines are allowed for use in poultry for prevention of disease and as growth promoters because they are not classified as important for humans [35]. Unfortunately, this study observed multiresistance by most isolates to a lot of these drugs that are prohibited for use (Table 3) in agreement with [36] in earlier work in Ghana and [19] in Nigeria. This finding suggests that recommendations by WHO [35] are not being complied with. Nondiscriminatory use of antibiotics in humans and animals results in the reduction of the effect of such antibiotics when bacteria are passed on to humans from animals. This could lead to treatment failure with possible repercussions to public health. Patterns of antibiotic resistances shown in human isolates in Accra by MOH/MOFA/MESTI/MFAD [36] are very similar to patterns observed in this study (Table 3). This may be a confirmation of the indiscriminate and unregulated use of antibiotics for both human and agriculture purposes.

Radiation processing at 5kGy, however, totally eliminated all microbes in samples making their microbial quality acceptable (Table 1) indicating radiation is very efficient even at lower doses in decontaminating poultry feed in agreement with [37]. Generally, gamma irradiation has been found to be capable of reducing the microbial load of dehydrated ingredients, spices, and dried herbal products effectively without negative attributes such as loss of heat-sensitive active ingredients and discoloration associated with conventional technologies such as the use of chemical preservatives, heat, and fumigants [11].

In conclusion, the current study found feed from the BNARI poultry unit to be contaminated with coliform counts above permissible limits and MDR food-borne pathogens suggesting a poor sanitary status and a public health risk. Low dose of radiation (5KGy) was however found to be effective in decontaminating poultry feed and eliminating possible associated food safety risks.

We recommend based on our conclusions that poultry feeds should be irradiated at 5 kGy after mixing and that further research should be conducted to ascertain appropriate packaging and storage after radiation. With respect to antibiotic resistance, we recommend a nationwide surveillance that should monitor the use of antibiotics in veterinary practice alongside education of farmers and veterinarians to curb misuse and overuse of antibiotics that will ultimately reduce resistant bacteria and antibiotic residue through the food chain.

## Figures and Tables

**Table 1:** Microbiological counts (log_10_ cfu/g) of compounded feed before and after gamma irradiation.

Parameters determined	^a^Permissible levels of contamination	Radiation Dose (KGy)				
		0	5	10	15	20

TVC	≤6. 48	5.98	NDT	NDT	NDT	NDT
CC	≤ 4.0	4.76	NDT	NDT	NDT	NDT
STC	≤ 4.0	2.89	NDT	NDT	NDT	NDT
SC	absence per gram	NDT	NDT	NDT	NDT	NDT
YMC	≤5. 3	4.57	NDT	NDT	NDT	NDT

Values are means of two replicate determinations, TVC: total viable count, CC: coliform count, STC: *Staphylococcus* count, SC: *Salmonella* count, YMC: yeast and mold count, and NDT: not detected

^a^Permissible levels of contamination [17].

**Table 2:** Occurrence of bacteria isolates identified in feed samples.

Identified Bacteria spp	Occurrence (%)

*Bacillus cereus*	27. 3% (3 out of 11)
*Enterobacter cloacae*	54.5 (6 out of 11)
*Klebsiella pneumoniae*	18.2 (2 out of 11)

**Table 3:** Antibiotic resistance patterns of 11 bacterial isolates from feed samples.

Antimicrobials	No. of Isolates Tested	Resistant N(%)	Intermediate N(%)	Sensitive N(%)

PEN., AMP., FLX.	11(100%)	11(100%)	0(0)	0(0)
ERY.	11(100%)	10(90.9%)	0(0)	1(9.09%)
TET.	11(100%)	8(72.7%)	0(0)	3(27.3%)
CRX.	11(100%)	7(66.6%)	0(0)	4(36.4%),
CTX.	11(100%)	5(45.4%)	0(0)	6(54.5%)
CHL., CTR	11(100%)	4(36.4%)	0(0)	7(66.6%)
GEN.	11(100%)	3(27.3%)	0(0)	8(72.7%)
COT.	11(100%)	2(18.2%)	0(0)	9(81.8%)

PEN= Penicillin, AMP= Ampicillin, FLX= Flucloxacillin, ERY= Erythromycin, TET=Tetracycline, COT= Cotrimoxazole, GEN=Gentamicin, CHL=Chloramphenicol, CTR= Ceftriaxone, CTX= Cefotaxime, and CRX= Cefuroxime.

## Data Availability

The microbial counts (cfu/g), antibiotic resistance, and radiation dose (KGy) data used to support the findings of this study are included within the article.
