# Corrigendum to “Microbial Food Safety Risk to Humans Associated with Poultry Feed: The Role of Irradiation”

**DOI:** 10.1155/2019/2780520

**Published:** 2019-05-02

**Authors:** Tahiru Mahami, Wellington Torgby-Tetteh, Delali Isaac Kottoh, Leticia Amoakoah Twum, Emmanuel Gasu, Sylvester Nana Yao Annan, Daniel Larbi, Isaac Adjei, Abraham Adu-Gyamfi

**Affiliations:** Biotechnology and Nuclear Agriculture Research Institute (BNARI), Ghana Atomic Energy Commission, P. O. Box LG 80, Legon, Accra, Ghana

In the article titled “Microbial Food Safety Risk to Humans Associated with Poultry Feed: The Role of Irradiation” [1], the name of the second author was given incorrectly as Wellington Togby-Tetteh. The author's name should have been written as Wellington Torgby-Tetteh. The revised authors' list is shown above.
